# The Value of a Complete Blood Count (CBC) for Sepsis Diagnosis and Prognosis

**DOI:** 10.3390/diagnostics11101881

**Published:** 2021-10-12

**Authors:** Luisa Agnello, Rosaria Vincenza Giglio, Giulia Bivona, Concetta Scazzone, Caterina Maria Gambino, Alessandro Iacona, Anna Maria Ciaccio, Bruna Lo Sasso, Marcello Ciaccio

**Affiliations:** 1 Department of Biomedicine, Neurosciences and Advanced Diagnostics, Institute of Clinical Biochemistry, Clinical Molecular Medicine and Clinical Laboratory Medicine, University of Palermo, 90127 Palermo, Italy; luisa.agnello@unipa.it (L.A.); rosaria.vincenza.giglio@alice.it (R.V.G.); giulia.bivona@unipa.it (G.B.); concetta.scazzone@unipa.it (C.S.); cmgambino@libero.it (C.M.G.); bruna.losasso@unipa.it (B.L.S.); 2 Department of Laboratory Medicine, University Hospital “P. Giaccone”, 90127 Palermo, Italy; alessandro.iacona84@gmail.com; 3 Unit of Clinical Biochemistry, University of Palermo, 90127 Palermo, Italy; annamaria.ciaccio@unipa.it

**Keywords:** biomarker, sepsis, CBC, CPD, thrombocytopenia, anemia, lymphocytes, neutrophils, monocytes, RBC

## Abstract

Sepsis represents an important global health burden due to its high mortality and morbidity. The rapid detection of sepsis is crucial in order to prevent adverse outcomes and reduce mortality. However, the diagnosis of sepsis is still challenging and many efforts have been made to identify reliable biomarkers. Unfortunately, many investigated biomarkers have several limitations that do not support their introduction in clinical practice, such as moderate diagnostic and prognostic accuracy, long turn-around time, and high-costs. Complete blood count represents instead a precious test that provides a wealth of information on individual health status. It can guide clinicians to early-identify patients at high risk of developing sepsis and to predict adverse outcomes. It has several advantages, being cheap, easy-to-perform, and available in all wards, from the emergency department to the intensive care unit. Noteworthy, it represents a first-level test and an alteration of its parameters must always be considered within the clinical context, and the eventual suspect of sepsis must be confirmed by more specific investigations. In this review, we describe the usefulness of basic and new complete blood count parameters as diagnostic and prognostic biomarkers of sepsis.

## 1. Introduction

Sepsis is a highly complex disease caused by the dysregulation of the host response to infection that leads to an uncontrolled inflammatory response followed by immunosuppression. It occurs as a complication of infections acquired both in the community and in healthcare settings, especially in intensive care units (ICU) [1], where it represents the most common cause of death, accounting for more than 50% of ICU mortality [2]. Overall, sepsis is considered a global health burden, with an important economic impact [3]. Thus, the rapid detection of sepsis is crucial in order to prevent adverse outcomes and reduce mortality by promptly starting the treatment before the occurrence of irreversible damage. It has been estimated that each hour of treatment delay is associated with a 7–10% increase in sepsis-related mortality [4]. However, the early diagnosis of sepsis is still challenging today because it is characterized by non-specific specific signs and symptoms. Thus, many efforts have been made to identify a reliable biomarker for screening patients at high risk of sepsis. Among all investigated sepsis biomarkers [5,6,7,8], parameters belonging to the complete blood count (CBC) could represent precious tools. Indeed, CBC has several advantages: (i) it represents the first-line laboratory test most commonly ordered in all clinical settings, from the emergency department (ED) to the ICU; (ii) clinicians routinely request CBC as part of the management of patients; (iii) it is easy to perform; (iv) it is cheap; (v) it has a fast turn-around time (TAT); (vi) it is available in all health facilities.

The aim of this review is to highlight the usefulness of CBC parameters for sepsis diagnosis and prognosis by putting together published literature on the subject matter. Specifically, we performed literature research on PubMed by combining the term “sepsis” and all terms related to the CBC parameters. We excluded articles written in other languages than English and those not related to sepsis.

## 2. Sepsis Definition and Pathogenesis

**Figure 1:**
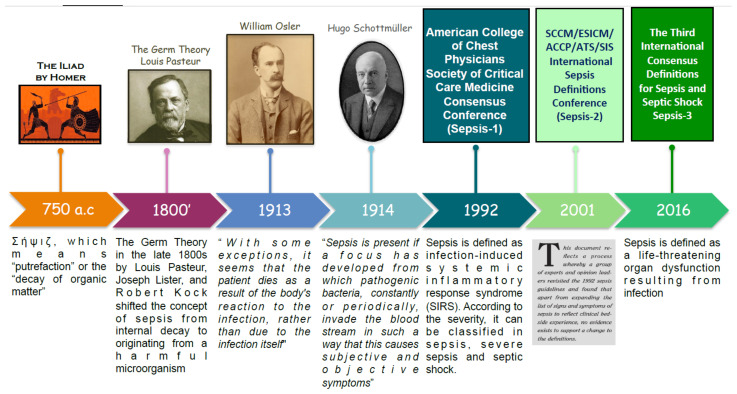
Timeline of the evolution of the sepsis definition.

Sepsis has a long history dating back to over 2700 years ago when it was first mentioned in the ancient Greek poems of Homer. Since then, the definition of sepsis has shifted over time (Figure 1). Originally sepsis was thought to be an internal rotting or decaying due to the smell of patients affected [9]. The development of medical hygiene and the germ theory in the late 1800s modified the concept of sepsis from internal decay to originating from a harmful microorganism. In 1913, William Osler noted “except on few occasions, the patient appears to die from the body’s response to infection rather than from it” [10]. The next year, Hugo Schottmüller created the basis for the modern definition of sepsis: “Sepsis is present if a focus has developed from which pathogenic bacteria, constantly or periodically, invade the bloodstream in such a way that this causes subjective and objective symptoms” [11]. However, over the course of the twentieth century, there emerged a need for an accurate and internationally recognized definition of sepsis. In 1992, the consensus conference of the Society of Critical Care Medicine and the American College of Chest Physicians, also known as Sepsis-1 conference, defined sepsis as infection-induced systemic inflammatory response syndrome (SIRS) [12]. Such definition was updated in 2002 by Sepsis-2 conference [13]. After two decades, in 2016, the Sepsis-3 conference revolutionized the definition of sepsis by removing the concept of SIRS and describing it as “a life-threatening organ dysfunction resulting from infection” [14]. In other words, sepsis is caused by a deregulated response to a pathogen.

Sepsis is a highly heterogeneous disease both in terms of etiology and pathogenesis [15]. Gram-negative bacteria are the most frequent pathogen causing sepsis, followed by gram-positive. Also, parasites and fungi can cause sepsis but to a lesser extent than bacteria. Notably, in about one-third of patients, the causative agent is not detectable.

The pathogenesis of sepsis can be ideally divided into two phases. An initial phase characterised by an intense inflammatory response to infection leading to the release of pro-inflammatory cytokines. Most patients (≈60%) recover, while 30% transit to the late phase, characterised by immunosuppression [16]. In this scenario, the immune response, which consists of the innate and adaptive systems, has a key role. The innate immune system represents the first line of defence against pathogens. It is not specific and acts rapidly to fight infection. Eosinophil, basophil, and phagocytic cells, including macrophages and neutrophils, are components of the innate immune system. The adaptive immune system, consisting of lymphocytes T and B, involves antigen-specific response, which is specific for the pathogen and regulated by crosstalk with innate immune cells. The adaptive immune response is important for confining the inflammation and tissue damage after infection and for returning immune homoeostasis.

During sepsis, pathogen-associated molecular patterns (PAMP) and danger-associated molecular patterns (DAMPs) induce the activation of innate immune cells, which release pro-inflammatory cytokines, leading to a robust inflammatory response characterized by the so-called “cytokine storm”. The excessive inflammatory response could induce cell and tissue damage, leading to multi-organ dysfunction. Additionally, during sepsis, the adaptive immune response is deregulated, leading to immune suppression, which promotes secondary infections.

## 3. Basic Complete Blood Count

The CBC is the most common and easy-to-perform laboratory test, which provides a wealth of information on individual health status. The appropriate interpretation of this test is pivotal for the early detection of several clinical conditions, which should be further investigated by laboratory and clinical analysis.

The CBC parameters can be grouped into three categories: (i) white blood cells (WBC), (ii) red blood cells (RBC), (iii) platelets. In this section, we describe the parameters included in a basic CBC, which can be provided by any hematological analyzer.

### 3.1. White Blood Cells

White blood cells, also known as leukocytes, are a heterogeneous population including lymphocytes, monocytes, and granulocytes, consisting of neutrophils, eosinophils, and basophils. It can be expressed as a percentage or as an absolute value. The WBC absolute value has clinical significance and is more informative than the relative one (percentage) because it indicates the medullary response to inflammatory stimuli. The relative value is helpful for evaluating which WBC population is mainly involved in the inflammatory process, allowing an etiological diagnosis.

Commonly, the increase of total WBC count is indicative of inflammation and infection. However, it can be altered in several clinical conditions, such as hemopathy, and in inflammatory non-infective disorders, such as rheumatoid arthritis, lupus, and malignancy [17,18]. Additionally, WBC count could be normal or even decreased in some cases of sepsis. Thus, total WBC has a poor specificity, which limits its usefulness as a biomarker of sepsis [14].

#### 3.1.1. Lymphocytes

Lymphocytes are key components of the adaptive immune response. They make up approximately 20–40% of the total leukocyte count.

A hallmark of sepsis is the simultaneous presence of pro-inflammatory and immunosuppressive alterations [19]. The latter are characterized by the early massive depletion of lymphocytes due to apoptosis. Studies on mice and humans revealed that sepsis-induced apoptosis is driven either by extrinsic or intrinsic pathways [20,21]. Additionally, post-mortem studies on patients with septic shock showed that apoptosis occurs in both circulating cells and in solid organs [22]. Overall, sepsis-induced apoptosis leads to lymphocytopenia.

Several authors showed that lymphocyte count decreases in the early phase of sepsis and that it is associated with poor outcomes [23,24,25,26]. Drewry et al. showed that persistent lymphocytopenia, defined as an absolute lymphocyte count ≤ 0.6 cells/μL × 103, on the fourth day after sepsis diagnosis, is associated with an increased risk of 28-day mortality [27]. Similarly, Chung et al. showed that severe basal lymphopenia is associated with an increased risk of death by day 28 in patients with septic shock admitted to ICU [28].

Also, Sheikh Motahar Vahedi et al. found that lymphocytopenia is a predictor of 28-day mortality in patients with sepsis admitted to the ED [29]. Finally, Hohlstein et al. showed that lymphocytopenia at ICU admission is associated with increased mortality [30].

Overall, literature evidence suggests that lymphocytopenia could be an indicator of increased risk of mortality in patients with sepsis.

#### 3.1.2. Monocytes

Monocytes represent the first line of defense against invading pathogens. They are activated by pattern recognition receptors (PRRs) and sepsis-associated hypoxia [31]. Monocytes control both the innate and adaptive immune responses to pathogens by different mechanisms, including phagocytosis; the release of reactive oxygen species, cytokines, and chemokines; the recruiting of neutrophils; antigen presentation; and the activation of lymphocytes [32].

Overall, monocytes can be classified into three different sub-population based on the different expression of a co-receptor to lipopolysaccharide (LPS), CD14, and CD16 receptor: classical (CD14+++CD16−), intermediate (CD14++ CD16+), and non-classical (CD14+ CD16++) [33], which present different morphological, functional, and phenotypical characteristics. Under physiological conditions, classical monocytes are the most represented sub-population, accounting for around 85% of total circulating monocytes; the intermediate ones account for around 5%; and the non-classical ones account for the remaining 10% [34]. During sepsis, monocytes undergo a shift from classical to intermediate and non-classical forms [35].

Although monocytes have a pivotal role in sepsis, the value of monocyte count for sepsis diagnosis and prognosis is controversial. Some authors reported monocytosis, defined as an increase of monocyte count, while others described monocytopenia associated with increased mortality [32,36]. Thus, the value of monocyte count is limited. Noteworthy, CBC parameters detecting monocytes’ alteration, such as monocyte distribution width (MDW) and neutrophil-to-monocyte ratio, have shown promising results, as described above.

#### 3.1.3. Neutrophils

Neutrophils represent the most prevalent leukocytes and the most abundant innate cell population in systemic circulation, making up about 40% to 70% of the total leukocyte count [37]. They are a key component of the innate immune system and act as sentinels to eliminate invading pathogens.

When infection occurs, neutrophils rapidly migrate to the site of infection and eliminate the invading pathogen by several mechanisms, including phagocytosis and oxidative bursts, neutrophils extracellular traps to execute microbial killing [38].

Under physiological conditions, neutrophils undergo apoptosis to maintain their homeostasis. However, during sepsis, neutrophils undergo several functional alterations, including reduced migration, altered antimicrobial activity, and delayed apoptosis, contributing to immune dysfunction and persistent inflammation [39,40]. Altogether, neutrophil alterations contribute to the worsening of sepsis and the development of secondary complications.

During infection, the neutrophil count increases considerably, and it is generally associated with the overall severity of the infection. However, in severe sepsis, the neutrophil apoptosis is delayed, limiting the usefulness of neutrophil count in some cases.

Noteworthy, the increase of neutrophils, also known as neutrophilia, can occur in response to a stressor, including physical and emotional stress, as well as smoking [41]. Also, chronic disorders, such as inflammatory bowel disease, rheumatic disease, and hepatitis, as well as congenital disorders, such as Down syndrome, are characterized by baseline neutrophilia [42]. Finally, paraphysiological conditions, such as pregnancy and obesity, could be associated with acute to chronic neutrophilia [43].

Thus, neutrophil count alone has a poor diagnostic and prognostic power for sepsis. Notable, the neutrophil-to-lymphocyte ratio (NLR) has emerged as a reliable sepsis biomarker, as described above.

#### 3.1.4. Eosinophils

Eosinophils represent 1–4% of circulating leukocytes and have a pivotal role in host defence against helminths, the propagation of allergic conditions, and immune and inflammatory networks. They possess receptors for many inflammatory mediators and produce and release an array of biologically active molecules, including cytotoxic proteins, lipid mediators, chemokines, and cytokines. Upon physiological or pathological stimuli, such as infection, eosinophils can migrate into target organs and tissues, where, once activated, they release their products and promote local inflammation, as well as tissue remodeling [44]. Eosinophils are recognized as an important player in modulating local and systemic immune and inflammatory responses.

The reduction of circulating eosinophil count, termed eosinopenia, in response to infection was firstly described in 1893 by Zappert et al. [45]. It is now well known that eosinopenia occurs during acute infection [46].

Abidi et al. firstly evaluated the potential role of eosinopenia as a biomarker of sepsis, showing that it has good sensitivity and specificity in diagnosing sepsis [47]. Then, several authors addressed the value of eosinopenia for diagnosing and predicting the prognosis of sepsis, achieving inconsistence and controversial results [48,49,50,51]. Notably, the value of eosinopenia as a criterion of sepsis has been the subject of debate for decades.

A recent meta-analysis by Lin et al., including a total of 3.842 patients, showed that eosinopenia has a high incidence in sepsis, but it is not superior to conventional biomarkers for diagnosing sepsis, such as C reactive protein (CRP) and procalcitonin (PCT) [52]. It should be considered that the studies currently available on the accuracy of eosinopenia as a sepsis biomarker present a high heterogeneity, with sensitivity ranging from 23.2 to 92.5% and specificity from 28.57 to 91%. However, the authors of the meta-analysis conclude that eosinopenia can still be used in clinical practice for detecting sepsis because it is a simple, convenient, fast, and inexpensive biomarker.

#### 3.1.5. Basophils

Basophils represent the rarest granulocytes, making up about 0.5–1% of the total leukocyte count. They are characterized by the presence of basophilic granules within the cytoplasm, containing several allergic mediators such as histamine, and a high-affinity IgE receptor on the cell surface. The physiological function of basophils has for many years remained enigmatic. Indeed, the study of basophils has long been hampered by both their rarity and the lack of tools for their detection and functional analyses. However, recently developed tools, such as basophil-depleting antibodies and engineered mice deficient for only basophils, shed light on the important role of basophils in allergic responses, protective immunity against parasitic infections and the regulation of the immune system [53]. Specifically, some authors showed that basophils have a role in initiating Th2 cell differentiation by acting as antigen-presenting cells [54,55]. Piliponsky et al. showed that basophils could enhance the innate immune response to bacterial infection and help prevent sepsis in an experimental model [56]. However, only one study explored the role of basophils in patients with sepsis. The authors showed that septic critically ill patients with decreased basophil count at admission to ICU had an increased mortality risk [57].

### 3.2. Red Blood Cells

Red blood cells, also termed erythrocytes, are the most abundant circulating cells and are produced within bone marrow through a complex and multi-step process, known as erythropoiesis, which begins with the differentiation of multipotent hematopoietic stem cells into erythroid-committed precursors. The final step leads to the production and release in the bloodstream of reticulocytes, which complete the maturation process into erythrocytes.

Under physiological conditions, RBCs have a characteristic biconcave disc shape, a lifespan of 120 days, and are metabolized by macrophages in the spleen and liver [58]. The best-known function of RBC is the transport and exchange of O_2_ and CO_2_ between the lungs and other tissues. However, they also have a pivotal role in cellular blood immunity, representing the main circulating bactericidal cells [59,60].

Sepsis is characterized by decreased RBC count, which could be due to several mechanisms related to the altered production or survival of RBC [61]. The suppression of RBC production could be the result of functional iron deficiency, decreased erythropoietin synthesis, infection, and inflammation [62]. Moreover, pre-existing clinical conditions, such as cancer, liver disease, or renal impairment as well as new-onset multiple organ dysfunction, particularly of hepatic and renal systems, may contribute to RBC loss during sepsis. Other contributing factors include disseminated intravascular coagulation (DIC), pathogen-associated hemolysis, hypoadrenalism, and nutritional deficiency. In addiction, the volume resuscitation-induced hemodilution is associated with decreased RBC count. Finally, blood loss can also occur by repeated phlebotomy, via the gastrointestinal tract, or from surgical procedures. Withdrawal of blood has been estimated to result in a mean daily loss of 24 to 41 mL of blood [62].

The shortening of RBC survival could be due to pathogen- and immune-reaction-induced RBC alteration. Indeed, sepsis dramatically alters RBC morphology and rheology (viscosity, aggregation, and deformability). As a consequence, altered RBCs are more rapidly cleared from the circulation through increased uptake by the reticuloendothelial system of the spleen and/or the liver [63]. Overall, reduced RBC count has no diagnostic or prognostic power for sepsis.

Basic CBC provides several parameters related to RBC characteristics, including hemoglobin (Hb), hematocrit, mean cell (or corpuscular) volume (MCV), mean corpuscular hemoglobin (MCH), mean cell hemoglobin concentration (MCHC), and red distribution width (RDW).

#### 3.2.1. Hemoglobin

Hb has a critical role in oxygen delivery to the tissues. Notably, the decrease of hemoglobin, defined as anemia, is common in patients with sepsis and overall in critical illness [64]. It has been estimated that two-thirds of patients admitted to ICU have Hb levels < 120 g/L and that about 40% have Hb < 100 g/L; 97% of patients develops anemia by day 8 and 100% by day 13 of ICU hospitalization.

The measurement of Hb concentration is pivotal for RBC transfusion decision-making. However, it is always important to evaluate if the benefits of additional oxygen-carrying capacity outweigh the risks. Indeed, in the presence of important organ hypoperfusion, an increased Hb concentration leading to increased oxygen delivery could exacerbate organ dysfunction and worsen the patients’ outcomes. The Surviving Sepsis Campaign 2016 recommends “RBC transfusion should occur only when hemoglobin concentration decreases to <7.0 g/L in adults in the absence of extenuating circumstances, such as myocardial ischemia, severe hypoxemia, or acute hemorrhage” [65].

#### 3.2.2. Hematocrit

Hematocrit indicates the fractional volume of a whole blood sample occupied by RBCs, expressed as a percentage.

Sepsis is characterized by a reduction of hematocrit. The value of hematocrit is used as a target for transfusion [66]. In patients with septic shock, targeting a hematocrit value of 30% in those with low central venous oxygen saturation during the first 6 h of resuscitation has been proposed [67,68].

#### 3.2.3. MCV

The MCV measures the average size and volume of the circulating RBC. In clinical practice, MCV is a useful index for classifying anemia as microcytic, normocytic or macrocytic. Sepsis is commonly characterized by normocytic anemia. MCV alone does not have a role as a sepsis biomarker. However, the combination of MCV with the standard deviation of erythrocyte volume for calculating RDW has been deeply investigated in patients with sepsis, as described above.

#### 3.2.4. MCH and MCHC

MCH and MCHC are measures of the hemoglobin content of RBC. MCH indicates the amount of hemoglobin per RBC, while MCHC expresses the amount of hemoglobin per unit volume. To date, the value of MCH and MCHC in patients with sepsis has never been explored.

#### 3.2.5. RDW

RDW represents a measure of the RBC anisocytosis, defined as the presence of highly heterogeneous RBCs in terms of size and volume [69].

Decreased RDW has no clinical implication, while increased values are indicative of large size variation of RBCs, and, consequently, are clinically meaningful. For a long time, RDW has been regarded as a biomarker for the differential diagnosis of thalassemia and iron-deficiency anemia [70]. In the last decades, a role for RDW in non-hematological disorders, such as autoimmune diseases, cardiovascular diseases, and critical illness, including sepsis, has emerged.

During sepsis, oxidative stress and inflammation, which are features of the sepsis cascade, lead to the reduction of survival and the suppression of the maturation of RBCs, resulting in the release of premature RBCs and consequently, in an increase of RDW [71]. Specifically, on one side, pro-inflammatory cytokines hamper erythropoietin-induced erythrocyte proliferation and maturation; on the other side, oxidative stress reduces RBC survival, producing large premature RBCs. Several authors showed that the rise of RDW (>15%) in septic patients is an independent predictor of long- and short-term adverse clinical outcomes, including mortality, especially in the ICU [72,73,74,75,76,77,78,79]. Additionally, Zhao et al. developed a nomogram based on the combination of CBC parameters, including RDW, to predict the risk of mortality in patients with sepsis admitted to the ED [80]. The nomogram, which included increased age, neutrophil-to-lymphocyte ratio (NLR), platelet-to-lymphocyte ratio (PLR), and RDW, as well as a decreased lymphocyte-to-monocyte ratio, showed good prognostic accuracy. Additionally, Chen et al. developed a clinical prediction rule, namely the CHARM score, based on clinical and laboratory parameters, including RDW [81]. The CHARM score showed good performance for predicting in-hospital mortality in patients with clinically suspected sepsis in the ED.

However, some authors failed to find an association between RDW and outcome in septic patients [82,83].

Overall, most of the evidence supports the role of RDW as an independent prognostic biomarker for sepsis [84].

To date, only a few studies evaluated the accuracy of RDW for diagnosing sepsis [85,86,87]. Zhang et al. [85] and Laukemann et al. [87] found that RDW was not a reliable biomarker for predicting sepsis. On the other hand, Park et al. showed that septic patients had significantly higher RDW values than healthy controls. Additionally, RDW displayed a high diagnostic accuracy for sepsis prediction.

Taken together, the literature evidence on the possible role of RDW as a diagnostic biomarker of sepsis is still poor, and further efforts are warranted.

### 3.3. Platelets

Platelets are the smallest elements in the bloodstream. They are anucleate cells produced mainly in the bone marrow by the fragmentation of the megakaryocyte extrusions into the vasculature. Beyond their well-known role in hemostasis, they contribute to the innate immune response to infection and inflammation. Platelets function as sentinels for the rapid detection of microbial invasion and orchestrate a complex intravascular immune defense response that protects against bacterial dissemination [88].

During sepsis, multiple factors, including the direct interaction of the pathogen with DAMP receptors expressed on the platelet surface, coagulation system activation, inflammatory response, and endothelial tissue damage, induce the activation of platelets. Upon activation, platelets exert several functions. Activated platelets rapidly aggregate and express multiple receptors on their surface that further enhance their aggregation with nearby platelets and leukocytes or that directly bind to and sequester extracellular pathogens [89]. Activated platelets also release microbicide molecules and chemokines that facilitate pathogen elimination, signal immune cells, and contribute to inflammation. Finally, platelets promote a pro-inflammatory phenotype of neutrophil [90,91], as well as monocyte, differentiation into macrophages.

The clinical monitoring of platelet count has emerged as a precious tool in the management of septic patients [92]. Indeed, several authors showed that platelet count is a useful diagnostic and prognostic biomarker in sepsis [88]. The reduction of platelet count, termed thrombocytopenia, is a common finding in septic patients, with an incidence ranging from 20 to 70% across studies [93]. The inclusion of platelet counts as a core parameter for calculating the sepsis-related organ dysfunction score (SOFA); the score emphasizes the importance of such CBC parameters [14]. A low platelet count is strongly correlated with adverse outcomes in sepsis patients, and it is often used for stratifying patients: mild thrombocytopenia (platelet count < 100–150 × 109/L), moderate thrombocytopenia (platelet count between 50 and 100 × 109/L), and severe thrombocytopenia (platelet count < 50 × 109/L), which is associated with worse outcomes [94].

Noteworthy, the kinetic of platelets provides important prognostic information and is more predictive for mortality than a single measurement. The failure of platelet counts to return into the normal range during acute illness is associated with increased mortality, while the recovery of platelet count is associated with survival to ICU discharge. Thus, not only the severity of the thrombocytopenia but especially its persistence is associated with worse outcomes [90].

Sepsis-associated thrombocytopenia is the result of several mechanisms, including the considerable consumption of circulating platelets, which are recruited from the circulation and sequestered within highly vascular organs, such as the lungs and liver; the decreased thrombopoiesis; the hemodilution; the platelet-leukocyte aggregation; the direct pathogen-induced thrombocytopenia; the immune-mediated destruction of platelets; drug-induced thrombocytopenia; DIC; and hemophagocytic lymphohistiocytosis [88,95,96].

Although sepsis is one of the most common causes of thrombocytopenia in critically ill patients, several other causes can mimic sepsis-related thrombocytopenia, such as myelodysplastic syndrome and aplastic anemia, as well as drugs, such as thiazide or chemotherapics, which inhibit platelet production [97].

Not only the platelet count but also platelet-derived indices have been evaluated as biomarkers of sepsis. Platelet indices include the platelet distribution width (PDW), which is a measure of platelet anisocytosis, which, accordingly, increases during accelerated platelet turnover; the mean platelet volume (MPV), which is an indicator of platelet size; and plateletcrit, a measure of total platelet mass. Some authors showed that platelet indices are reliable prognostic biomarkers of sepsis, whereas others found the opposite [39,96,98]. To date, there are few studies to draw a conclusion on the potential role of platelet indices as biomarkers of sepsis.

## 4. CBC Parameters Ratios

### 4.1. Neutrophil-to-Lymphocyte Ratio

The neutrophil-to-lymphocyte ratio (NLR) is calculated as the neutrophil count divided by the lymphocyte count. During sepsis, neutrophils and lymphocytes rapidly respond to microbial infection in a different manner. Neutrophil count increases dramatically, while the lymphocyte count decreases. Although neutrophil count alone is associated with the overall severity of the infection/inflammation, neutrophil apoptosis is delayed in complicated sepsis cases, suggesting that it has limited prognostic value in some cases [99]. Changes in NLR are indicative of the balance between neutrophil and lymphocyte counts. Several authors showed that NLR is an early biomarker of sepsis, regardless of the source of sepsis, and correlates with sepsis severity scores, such as The Acute Physiology and Chronic Health Evaluation II (APACHE II) score and SOFA score [100,101,102,103,104,105]. Noteworthy, NLR increases rapidly following infection.

A recent meta-analysis of 14 studies, including 11,564 septic patients, revealed that NLR is a reliable prognostic indicator in patients with sepsis [106]. Specifically, non-survivor patients showed significantly higher levels of NLR than survivors. Thus, NLR is an independent predictor of worse outcomes [101]. It seems to be a more reliable sepsis biomarker than either neutrophil count or lymphocyte count alone [107]. When interpreting NLR, it should be kept in mind that its levels can also increase in other conditions, such as hypovolemic shock or exogenous steroid therapy [108].

Overall, increased NLR levels are independently associated with adverse prognosis in septic patients. Unfortunately, several decisional NLR cut-off values have been proposed, ranging from 4.36 to 23.8 [106], independently based on the hematology analyzer used. Thus, before introducing it in clinical practice, a univocal cut-off should be validated.

### 4.2. Monocyte-to-Lymphocyte Ratio, Platelet-to-Lymphocyte Ratio, and Mean Platelet Volume-to-Platelet Count

Literature data regarding the role of monocyte-to-lymphocyte ratio (MLR), platelet-to-lymphocyte ratio (PLR), and platelet count-to-mean platelet volume (PC/MPV) in patients with sepsis are scarce. Djordjevic et al. assessed such ratios in patients with sepsis, showing that PC/MPV was higher in non-survivors than survivors, while MLR and PLR did not differ significantly [109]. Similarly, Oh et al. showed that a high PC/MPV ratio (>3.71) was an independent predictor of 28-day mortality [110]. Shen et al. found a strong association between increased PLR and hospital mortality in a large observational study including 5537 sepsis patients [111]. On the other hand, Liberski et al. [98], as well as Ates et al. [112], found that PLR, MLR, and PLT/MPV were not reliable biomarkers for sepsis screening or prognostication. Thus, it is not possible to draw conclusions on the usefulness of these ratios in patients with sepsis and further studies are warranted.

## 5. Cell Population Data

Over the past few decades, hematology analyzers have undergone important technological advancements. The new generation of instruments can generate the so-called cell population data (CPD), which provides quantitative information on the morphological and functional characteristics of blood cells. CPD can be generated by two different technologies, VSC technology and fluorescence flow cytometry.

VSC technology uses direct current impedance for measuring the volume (V) of the cells, a laser beam to measure multiple angled light scatters (S) for evaluating cytoplasmic granularity and the nuclear structure of the cells, and radiofrequency conductivity (C) to analyze the cytoplasmic composition and nuclear volume of the cells [113].

Fluorescence flow cytometry is based on the use of blood-cell membrane surfactant reagents and the fluorescent labeling of cell epitopes followed by flow cytometry analysis. Such technology provides information on cell size, internal complexity (granularity), and the content of nucleic acids (DNA and RNA). Briefly, the differential leucocyte count is based on the criteria of granularity (side scatter light), cell size (forward scatter light), and nucleic acid/protein content of cells (fluorescent light intensity). The optical signals of leucocyte differential are presented in the three axes of the white blood cells differential fluorescence (WDF) channel scattergram [113].

CPD parameters provide quantitative values of volume, granularity, and complexity for each cell. The changes in CPD values can provide precious information on the morphological and functional transformation of cells in response to an infection. Thus, several CPD parameters have been evaluated as a biomarker of sepsis. In this section, we describe the most promising CPD parameters for sepsis.

### 5.1. Monocyte Distribution Width

The monocyte distribution width (MDW), also named early sepsis indicator (ESId), represents a measure of the monocytes’ anisocytosis. It has been approved by the Food and Drug Administration (FDA) as a biomarker for the early detection of sepsis in ED. Recently, several authors showed that MDW has good diagnostic accuracy for early-identifying patients at high risk of developing sepsis, especially in the ED and the ICU [114,115,116,117,118,119,120,121,122,123]. MDW is characterized by a high negative predictive value for sepsis diagnosis. Thus, a value under the decisional cut-off allows the reliable exclusion of the presence of sepsis. In clinical practice, MDW could represent a red flag to identify patients that should undergo further clinical and laboratory evaluations to confirm the suspicion of sepsis. However, further efforts are required before introducing it in clinical practice. Indeed, there is a high heterogeneity among studies on the optimal MDW decisional cut-off value, ranging from 20 to 27. This could be due to the difference in the study design, the different clinical settings in which the studies were performed (ED, ICU, or the infectious disease unit), the different method of calculations, or the type of anticoagulant used for the blood collection (K3-EDTA vs. K2-EDTA). Specifically, K2-EDTA anticoagulated whole blood samples were associated with lower MDW levels than K3-EDTA. Thus, different cut-off values for K2- or K3-EDTA anticoagulants must be used.

### 5.2. Mean Neutrophil Volume and Mean Monocyte Volume

The mean neutrophil volume (MNV) and mean monocyte volume (MMV) represent the average size of the circulating neutrophil and monocyte populations, respectively. Several authors evaluated the role of MNV and MMV as biomarkers of sepsis, achieving encouraging results [124,125,126,127]. Arora et al. [117] found that MNV and MMV were increased in patients with sepsis compared to controls. Additionally, a significant decrease in MNV and MMV values was observed after the initiation of antibiotic therapy. Similarly, Mammen et al. showed that MNV and MMV were increased in patients with sepsis compared to non-septic patients admitted to ICU [126]. Noteworthy, both CPD parameters showed good diagnostic accuracy for sepsis, superior to traditional biomarkers, such as CRP and PCT. The best cut-off for detecting sepsis is 150 and 170 for MNV and MMV, respectively.

Overall, in critically ill patients with suspected sepsis, MNV and MMV may help strengthen the diagnostic probability of sepsis. Further studies to validate the usefulness of such biomarkers are necessary to introduce them in clinical practice.

### 5.3. Neutrophil Fluorescence Intensity and Monocyte Internal Structure

Neutrophil fluorescence intensity (NE-SFL) and monocyte internal structure (MO-X) are two CPD parameters measured by fluorescence flow cytometry technology. Some authors found that both NE-SFL and MO-X have good diagnostic accuracy for diagnosing sepsis and correlate with disease severity [128,129,130].

### 5.4. Immature Granulocytes

Microbial pathogens stimulate the production of cytokines, which induce the release of immature granulocytes (IG) from the bone marrow, including promyelocytes, metamyelocytes, and myelocytes. In the peripheral blood, these immature granulocytes are an indicator of leukopoiesis and can be seen as bands, which are usually referred to as “left shift”. The granulocytic shift to the left reflects the active bone marrow response to infection. The band count is commonly obtained by a manual differential count, and, consequently, it is characterized by several drawbacks, such as long TAT; high interobserver variability; and low accuracy, precision and reproducibility. However, these limitations can be overcome by the quantification of IG on the last generation of automated hematology analyzers [131].

Severe sepsis is characterized by a marked increase, up to 10-fold, in neutrophil production by bone marrow and, consequently, by a rise in circulating immature neutrophils. Thus, IG has been assessed as a biomarker for sepsis diagnosis, achieving contradictory results. Some authors found that an elevated value of IG is indicative of sepsis [108], while low values can reliably rule out sepsis [132]. Others reported IG to be nearly worthless [133]. Additionally, a high heterogeneity among studies exists, especially for optimal cut-off value, which ranges from 0.2% to 3% [108]. Thus, further studies are required to validate the usefulness of IG for sepsis diagnosis and to identify a univocal cut-off value.

### 5.5. Immature Platelet Fraction

The immature platelet fraction (IPF) is a CPD parameter obtained by flow cytometry technology. It reflects the number of circulating reticulated platelets, which represent the immature platelets and, consequently, provides a direct measure of the platelet production. It increases when platelet production rises and decreases when production falls. A recent systematic review showed that IPF is a reliable biomarker for sepsis diagnosis and prognosis, in terms of severity and mortality [134]. Indeed, it tends to increase before the onset of sepsis and correlates with the scores conventionally used for assessing the severity of sepsis, such as the SOFA score.

### 5.6. Delta Neutrophil Index

The delta neutrophil index (DNI) is an indicator of the circulating immature granulocytes. Some authors showed that DNI has a prognostic value in patients with sepsis. Specifically, increased levels of DNI are associated with mortality [135]. Additionally, Kim et al. found that a value of DNI constantly increased after 72 h treatment is associated with a worse prognosis [136]. Celik et al. showed that after 6–10 days of effective therapy, patients have normal levels of DNI [137]. Thus, DNI could also be useful for monitoring the efficacy of therapy.

## 6. Discussion

Sepsis is a complex disease, which still represents an open challenge worldwide. The early diagnosis of sepsis combined with appropriate management in the first hour of hospital admission is fundamental for patients with infections, ideally before any signs and symptoms of organ failure have appeared. However, the timely diagnosis of sepsis represents an ongoing challenge for any clinician. Actually, it is based on a combination of clinical and laboratory findings. Among the latter, the gold standard remains the blood culture, but it has several drawbacks, including a long TAT, a high rate of false-negatives, and the vulnerability to pre-analytical variables [138]. Additionally, CRP and PCT are the most common required biomarkers for sepsis diagnosis, prognosis, and therapeutic decision-making [139,140,141,142,143]. Nonetheless, they are characterized by suboptimal diagnostic accuracy, low specificity, modest sensitivity, and high cost, especially for PCT. The attention to the early diagnosis of sepsis has fueled interest in identifying low-cost biomarkers available routinely. In this scenario, CBC parameters are ideal biomarkers.

They represent early, rapid, inexpensive, and widely available biomarkers, allowing efficient and timely patient management by promptly detecting patients at high risk of sepsis, also when the clinician does not suspect it, and by allowing a strengthened monitoring and more aggressive treatment.

**Figure 2:**
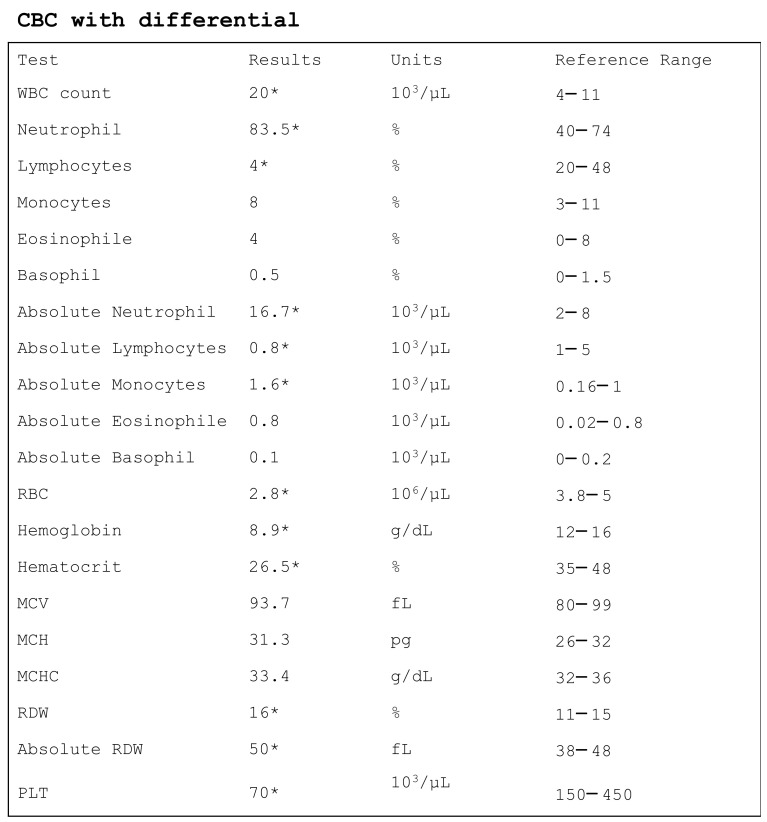
The CBC of an adult patient with sepsis. WBC, white blood cells; RBC, red blood cells; MCV, mean cell volume; MCH, mean corpuscular hemoglobin, MCHC, mean cell hemoglobin concentration, RDW, red distribution width; PLT, platelet. * indicates values out of reference range. Notably, reference ranges change according to the instrument used.

The CBC of a patient with sepsis is commonly characterized by lymphocytopenia, neutrophilia, eosinopenia, thrombocytopenia, increased RDW, and increased NLR (Figure 2). The importance of thrombocytopenia in patients with sepsis is emphasized by the inclusion of platelet count in the SOFA score. Moreover, anemia, detected by a reduction of hemoglobin and hematocrit, is a common finding in patients with sepsis. Accordingly, both hematocrit and hemoglobin values are used to guide and monitor blood transfusion therapy. Finally, CPD parameters, which are generated by the new generation analyzers, have been recently evaluated as potential biomarkers of sepsis. Among these, literature data support the usefulness and reliability of MDW and IPF as biomarkers for early sepsis screening and prognosis in acute settings. Although CPD parameters have the great advantage of being available together with basic CBC, they present some important limitations, which hamper their introduction in clinical practice. Indeed, they are not available on all instrumentations, and they depend on the optical design of the analyzer used. Consequently, there is a lack of harmonization among different instruments.

**Figure 3:**
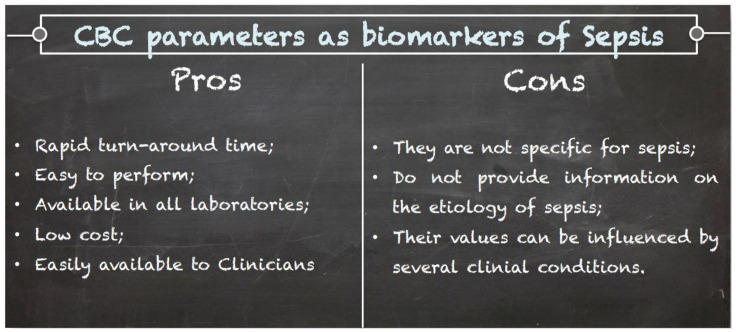
Pros and cons of CBC parameters as biomarkers of sepsis.

Overall, an alteration of some CBC parameters should be interpreted by clinicians as a warning, which should raise the suspicion of sepsis, that must be always confirmed by more specific laboratory and clinical investigations. Notably, care must be taken when considering CBC parameters because most of them can be altered in many clinical conditions, and thus, the findings of CBC must always be considered within the clinical context (Figure 3). The clinical usefulness of CBC parameters is summarized in Table 1.

## 7. Conclusions

Global efforts have been made to reduce the burden of sepsis. Predicting sepsis could reduce healthcare costs and save patients’ lives by avoiding multi-organ dysfunction processes, reducing ICU admissions, and improving patients’ prognoses. To date, the ideal biomarker of sepsis has not been identified, and it is likely that it does not exist because sepsis is a very complex and heterogeneous disease [144]. Thus, a careful and integrated evaluation based on laboratory and clinical findings can help clinicians in its early recognition. CBC parameters detain great potential. Specifically, alteration of CBC parameters could represent an alert for clinicians, which should confirm the suspicion of sepsis with more specific laboratory and clinical investigations. Additionally, CBC parameters could assist clinicians in defining the severity of sepsis and monitoring the therapy.

## Figures and Tables

**Table 1:** Clinical usefulness of CBC parameters for sepsis.

	Parameter	Alteration	Clinical Usefulness
Basic	WBC	↑	Diagnosis
	Neutrophils	↑	Prognosis
	Lymphocytes	↓	Prognosis
	Monocytes	↑↓	Controversial
	Eosinophil	↓	Diagnosis
	Basophil	↓	Prognosis
	RBC	↓	None
	Hemoglobin	↓	Guide RBC transfusion
	Hematocrit	↓	Target for RBC transfusion
	MCV	-	-
	MCH	-	-
	MCHC	-	-
	RDW	↑	Prognosis
	Platelets	↓	Diagnosis and prognosis
CPD	MDW	↑	Diagnosis
	MNV	↑	Diagnosis
	MMV	↑	Diagnosis
	NE-SFL	↑	Diagnosis and prognosis
	MO-X	↑	Diagnosis and prognosis
	IPF	↑	Diagnosis and prognosis
	DNI	↑	Prognosis and monitoring therapy

WBC, white blood cells; RBC, red blood cells; MCV, mean cell volume; MCH, mean corpuscular hemoglobin, MCHC, mean cell hemoglobin concentration, RDW, red distribution width; MDW, monocyte distribution width; MNV, mean neutrophil volume; MMV, mean monocyte volume; NE-SFL, neutrophil fluorescence intensity; MO-X, monocyte internal structure; IPF, immature platelet function; DNI, delta neutrophil index.
